# Differentiating Laryngeal Carcinomas from Precursor Lesions by Diffusion-Weighted Magnetic Resonance Imaging at 3.0 T: A Preliminary Study

**DOI:** 10.1371/journal.pone.0068622

**Published:** 2013-07-09

**Authors:** De-Sheng Shang, Ling-Xiang Ruan, Shui-Hong Zhou, Yang-Yang Bao, Ke-Jia Cheng, Qin-Ying Wang

**Affiliations:** 1 Department of Radiology, The First Affiliated Hospital, College of Medicine, Zhejiang University, Hangzhou, Zhejiang Province, China; 2 Department of Otolaryngology, The First Affiliated Hospital, College of Medicine, Zhejiang University, Hangzhou, Zhejiang Province,China; NIH, United States of America

## Abstract

**Background:**

Diffusion-weighted magnetic resonance imaging (DWI) has been introduced in head and neck cancers. Due to limitations in the performance of laryngeal DWI, including the complex anatomical structure of the larynx leading to susceptibility effects, the value of DWI in differentiating benign from malignant laryngeal lesions has largely been ignored. We assessed whether a threshold for the apparent diffusion coefficient (ADC) was useful in differentiating preoperative laryngeal carcinomas from precursor lesions by turbo spin-echo (TSE) DWI and 3.0-T magnetic resonance.

**Methods:**

We evaluated DWI and the ADC value in 33 pathologically proven laryngeal carcinomas and 17 precancerous lesions.

**Results:**

The sensitivity, specificity, and accuracy were 81.8%, 64.7%, 76.0% by laryngostroboscopy, respectively. The sensitivity, specificity, and accuracy of conventional magnetic resonance imaging were 90.9%, 76.5%, 86.0%, respectively. Qualitative DWI analysis produced sensitivity, specificity, and accuracy values of 100.0, 88.2, and 96.0%, respectively. The ADC values were lower for patients with laryngeal carcinoma (mean 1.195±0.32×10^−3^ mm^2^/s) versus those with laryngeal precancerous lesions (mean 1.780±0.32×10^−3^ mm^2^/s; P<0.001). ROC analysis showed that the area under the curve was 0.956 and the optimum threshold for the ADC was 1.455×10^−3^ mm^2^/s, resulting in a sensitivity of 94.1%, a specificity of 90.9%, and an accuracy of 92.9%.

**Conclusions:**

Despite some limitations, including the small number of laryngeal carcinomas included, DWI may detect changes in tumor size and shape before they are visible by laryngostroboscopy. The ADC values were lower for patients with laryngeal carcinoma than for those with laryngeal precancerous lesions. The proposed cutoff for the ADC may help distinguish laryngeal carcinomas from laryngeal precancerous lesions.

## Introduction

Laryngeal squamous cell carcinoma (SCC) is one of the most common head and neck cancers, typically developing from precancerous lesions [1]. A precise preoperative diagnosis and assessment of T stage are important for making a prognosis in laryngeal carcinoma. Techniques such as contact laryngoscopy and fluorescence endoscopy can provide useful diagnostic information to differentiate laryngeal carcinomas from precancerous lesions and benign lesions[2]–[4]. However, these methods can only provide an accurate assessment of the surface extent of the tumor and are always performed under general anesthesia [2], [4]. Although magnetic resonance imaging (MRI) has been used to stage laryngeal carcinomas[5]–[6], studies have shown that differentiating laryngeal carcinomas from precancerous lesions and benign tumors is often difficult[7]–[8].

Recently, diffusion-weighted MRI (DWI) was introduced in head and neck cancers for differential diagnosis^9–16^, monitoring of the treatment response [9], [17], the differentiation of recurrence, and post-therapeutic changes after radiotherapy [9]. DWI uses the movement of water molecules to produce images that indirectly reflect information regarding cell density and microstructures in living tissues [18].These changes can be estimated and quantified in terms of the apparent diffusion coefficient (ADC) [18], [19]. DWI can detect changes in tumor size and shape before they are visible to the naked eye [18].

However, because there are limitations in the performance of laryngeal DWI, including the complex anatomical structure of the larynx causing susceptibility effects, the value of DWI in differentiating benign from malignant laryngeal lesions has only been investigated in a few studies[20]–[23]. Most of these studies reported some value in detecting recurrent tumors after radiotherapy/chemotherapy [20], [22], [23]. Today, these limitations have gradually been overcome due to steady improvements in MRI [15], [19], [20], driven by the wide variety of potential applications [19]. In head and neck cancers, previous studies confirmed a significant difference in the ADC between benign and malignant lesions by 3.0-T [15]and 1.5-T [24] MR.

An echo-planar sequence is the traditional choice for DWI, and it readily yield images with a low signal-to-noise ratio and strong susceptibility artifacts and distortion[19], [25]–[27]. A turbo spin-echo (TSE)-based DW sequence does not suffer from susceptibility artifacts[19], [25]–[27]. At our hospital, TSE DWI and linear regression with gradient b-values of 0 and 1,000 s/mm^2^ for ADC measurement are used.

To our knowledge, there is no previous report of using ADC measurements for laryngeal lesions with TSE DWI with 3.0-T MR to differentiate malignant from benign lesions. Thus, the purpose of the present study was to determine whether a threshold ADC value may help differentiate laryngeal carcinomas from precursor lesions by TSE DWI with 3.0-T MR, and to make a comparison with laryngostroboscopic findings.

## Materials and Methods

### 1. Approval

The institutional review board of The First Affiliated Hospital, College of Medicine, Zhejiang University (Hangzhou, China), approved the present study. Written informed consent was obtained from each patient before inclusion.

### 2. Patients

From November 2010 to November 2012, patients with laryngeal lesions who underwent preoperative laryngostroboscopy (Endo-stroboscope L, Atomos, Germany) and MRI were considered for inclusion in the study. The entry criteria were: (1) the patients were suspected of having precancerous or cancerous lesions of the larynx by laryngostroboscopy, (2) the patients had undergone 3.0-T MR (including DWI, b  = 0 or 1,000 s/mm^2^) before treatment, and (3) the patients underwent surgery and their diagnoses were confirmed by pathology (including frozen sections and routine pathological results).

Consequently, we included 56 patients (48 males and 8 females). Six were excluded due to susceptibility artifacts (due to linear blurring, geometric distortion, or imaging distortion) that compromised image quality. In the remaining 50 patients, the pathological results showed 33 laryngeal carcinomas (Table 1) and 17 precancerous lesions.

**Table 1:** Characteristics of patients and laryngeal lesions

Pt	Age/Sex	symptom	Preoperative diagnosis of laryngostroboscopy	Site	pTNM	Preoperative diagnosis of DWI	Pathological result	Treatment
1	68/M	Hoarseness and right neck mass 6 months	Bilateral VC edema and mass in subglottic area, motion of bilateral was normal	Subglottis	T_2_N_2_M_1_	LC(bilateral VC and anterior commissure	SCC in the subglottic area, metastasizing to rib	CRT
2	77/M	Hoarseness 1 year	1.5 cm×2.0 cm mass in left laryngeal ventricle, the motion of left VC was weak	Supraglottis	T_2_N_0_M_0_	LC(invasion into left supraglottis, glottis and subglottis)	SCC	PL
3	61/M	Hoarseness one month	0.5 cm×0.8 cm irregular mass in the anterior of bilateral VC and anterior commissure, the motion of bilateral was normal	Glottis	T_1b_N_0_M_0_	LC (involving in the anterior of bilateral VC)	SCC in the anterior of bilateral VC	PL
4	74/M	Hoarseness 6 months	1.2 cm×0.9 cm mass in left laryngeal ventricle, bilateral VC, motion of left VC was weak	Glottis	T_2_N_0_M_0_	LC(1.2×0.9 cm mass in the left VC	SCC(1.8×0.8 cm cancer in the left VC)	TL
5	49/M	Hoarseness 2 months	1.2 cm×0.9 cm mass in left laryngeal ventricle, bilateral VC, motion of left VC was weak	Glottis	T_1_N_0_M_0_	LC(confined to the left VC)	SCC	PL
6	66/M	Hoarseness one year	0.8 cm×1.0 cm in the left VC	Glottis	T_2_N_0_M_0_	LC in the left VC	SCC	Biopsy
7	66/M	Recurrent hoarseness one year, progressively twenty days	0.5 cm×0.3 cm in the left VC, motion of bilateral VC was normal	Glottis	T_1_N_0_M_0_	LC in the left VC	SCC	PL
8	61/M	Hoarseness 3 months	The left VC was rough, the motion of bilateral was normal	Glottis	T_1_N_0_M_0_	LC in the left VC	SCC in the left VC	PL
9	57/M	Hoarseness3 years, progressively 3 months	Mass in the bilateral VC	Glottis	T_2_N_0_M_0_	LC in the bilateral VC and anterior of commissure	SCC in the bilateral VC	PL
10	68/M	Hoarseness and pharyngalgia 3 months,companying many chronic diseases	0.5 cm×1 cm cauliflower-like mass in the anterior of left VC, the motion of bilateral was normal	Supraglottis	T_2_N_0_M_0_	LC(supraglottic tumor, glottis area is normal )	SCC(location in supraglottic area, invasion of bilateral VC epiglottic cartilage and thyroid cartilage)	TL+ND+ posteroperative RT
11	49/M	Hoarseness 2 months, progressively half month	1.8 cm×1.2 cm mass in the right laryngeal ventricle, the motion of bilateral was normal	Supraglottis	T_1_N_0_M_0_	LC(right laryngeal ventricle and right VC)	SCC(only in the right laryngeal ventricle)	PL+ND
12	56/M	Hoarseness 3 months	The anterior of the right VC was rough, the motion of bilateral was normal	Glottis	T_1_N_0_M_0_	LC in the right VC	SCC in the right VC	PL
13	70/M	Hoarseness 6 months	0.5 cm×0.8 cm mass in the right VC, 0.8 cm×1.5 cm mass in the left VC, the motion of bilateral VC was normal	Glottis	T_2_N_0_M_0_	LC(invasion of bilateral VC, arytenoids cartilage)	SCC(invasion of bilateral VC)	TL
14	74/M	Hoarseness 2 months	0.5 cm×0.8 cm mass in the right VC, 0.8 cm×1.5 cm mass in the left VC, the motion of bilateral VC was normal	Glottis	T_1_N_0_M_0_	LC	SCC(Right vocal cord)	PL
15	65/M	Recurrent hoarseness 4 years, progressively 3 months	5 mm×6 mm mass in the left VC and rough mucosa in the anterior right vocal cord, normal bilateral VC motion	Glottis	T_1_N_0_M_0_	Left VC carcinoma, right VC edema ( Fig. 3 )	Left VC SCC, right VC mild dysplasia	PL
16	68/F	Hoarseness one year	Rough mucosa in the anterior right VC, normal motion VC	Glottis	T_1_N_0_M_0_	Left VC carcinoma	Left VC papilloma canceration	PL
17	68/M	Hoarseness 2 months	Edema in bilateral VC and white mass in the right VC, the motion of bilateral VC was normal	Glottis	T_1_N_0_M_0_	LC in the right VC	First biopsy :right VC potential canceration, Second biopsy: right VC SCC	PL
18	57/M	Found mass in the larynx two days, radical resection of esophageal cancer 8 months ago	0.5 cm×0.5 cm mass in the right tongue surface of epiglottis, the motion of right VC was normal and the left VC was paralysis.	Supraglottis	T_1_N_0_M_0_	LC in the tongue surface of epiglottis, asymmetry in the bilateral VC	SCC in the epiglottis	PL+ND
19	51/M	Odynophagia,hoarseness 6 months	1.5×1.5 cm mass in the left aryepiglottic fold, the motion of bilateral VC was normal	Supraglottis	T_2_N_2a_M_0_	LC in the left aryepiglottic fold	SCC in the epiglottic,left lymph node metastasis	PL+left ND
20	67/M	Hoarseness 1 year	0.6×1.2 cm mass in the right VC, the motion of bilateral VC was normal	Glottis	T_2_N_0_M_0_	LC in the right VC, involved in right laryngeal ventricle	SCC	PT
21	68/M	Hoarseness 3 months	0.5×0.8 cm mass in the right VC, the motion of bilateral VC was normal	Glottis	T_1b_N_0_M_0_	LC in the right VC, involved in anterior commissure	SCC in the right VC, involved in anterior commissure	PL
22	80/M	Sound vague 7 months, discomfort in swallowing 2 months	1.0×1.0 cm mass in the left tongue surface of epiglottis, the motion of bilateral VC was normal	Supraglottis	T_1_N_0_M_0_	LC in the tongue surface of epiglottis	SCC	PL
23	59/M	Pharyngalgia 4 months	2.0×2.0 cm cauliflower-like mass in the tongue surface of epiglottis, the motion of bilateral VC was normal	Supraglottis	T_1_N_1_M_0_	LC in the epiglottis	SCC	PL
24	67/M	Laryngalgia 3 months	2.5×2.8 cm mass in the laryngeal surface of epiglottis, invading to left false VC, left laryngeal ventricle and left VC, the motion of left VC was weaken and the right was normal.	Supraglottis	T_2_N_0_M_0_	LC in the left hemilarynx	SCC in the epiglottis, left false VC, left laryngeal ventricle and left VC,	TL+left ND
25	57/M	Hoarseness 3 months	0.3×0.5 cm mass in the right VC, the motion of right VC was weaken and the left VC was normal	Glottis	T_1_N_0_M_0_	Right VC carcinoma	Early canceration	PT
26	55/M	Hoarseness 4 years	0.4×0.5 cm mass in the left VC, the motion of bilateral VC was normal	Glottis	T_1_N_0_M_0_	Left VC carcinoma	SCC in the left VC	PT
27	61/M	Hoarseness 1 month	0.5×0.8 cm mass in anterior of the left VC, anterior of the right VC and in the anterior commissure, the motion of bilateral VC was normal	Glottis	T_1b_N_0_M_0_	LC in the bilateral VC and laryngeal ventricle	SCC in the anterior of bilateral VC	PT
28	55/M	Hoarseness 2 months	0.4×0.6 cm mass in the right VC	Glottis	T_1_N_0_M_0_	LC in the right VC	SCC in the right VC	PT
29	56/M	Hoarseness 1 month	1.0×1.0 cm mass in the left laryngeal ventricle, the motion of bilateral VC was normal	Glottis	T_2_N_0_M_0_	LC in the left VC, involving laryngeal ventricle	SCC in the left VC, invading to the left laryngeal ventricle	PT
30	67/M	Hoarseness 1 month	0.5×0.8 cm mass in the left VC, the motion of bilateral VC was normal	Glottis	T_1_N_0_M_0_	LC in the left VC	SCC in the left VC	PT
31	56/M	Hoarseness 3 month	0.5×0.6 cm mass in the anterior commissure, the motion of bilateral VC was normal	Glottis	T_1b_N_0_M_0_	LC in the right VC, involving the anterior commissure	SCC in the right VC and the anterior commissure	PT
32	69/M	Hoarseness 1 year	1.0×1.5 cm mass in the right VC, the motion of the right was weaken and the left VC was normal	Glottis	T_2_N_0_M_0_	LC in the right VC, involving to the right false VC and right subglottic area	SCC in the right VC	PT
33	74/M	Hoarseness 1 year	1.2×1.5 cm mass in the right VC and anterior commissure the motion of bilateral VC was normal	Glottis	T_2_N_0_M_0_	LC in the right VC	SCC in the right VC	PT

Pt: patient; LC: laryngeal carcinoma; SCC: squamous cell carcinoma; CRT: concurrent chemoradiotherapy; TL: total laryngectomy; RT: radiotherapy; PL: partial laryngectomy; ND: neck dissection; COPD: chronic obstructive pulmonary disease; VC: vocal cord; pTNM: pathological TNM stage.

The English in this document has been checked by at least two professional editors, both native speakers of English. For a certificate, please see:

http://www.textcheck.com/certificate/DyMHuD.

### 3. MRI

MRI was performed on a 3.0-T MR unit (Achieva 3.0T; Philips Medical Systems, Best, The Netherlands) using a SENSE Neurovascular 16 coil. Conventional MRI included an axial T1-weighted TSE sequence with the following parameters: slice thickness, 4 mm; 24 slices; intersection gap, 1 mm; repetition time/echo time (TR/TE), 450 ms/10 ms; matrix, 320×224; field of view (FOV), 240×180 mm; and an axial T2-weighted TSE sequence (slice thickness, 4 mm; 24 slices; intersection gap, 1 mm; TR/TE, 4,000 ms/100 ms; matrix, 320×224; FOV, 240×180 mm). The coronal T2-weighted TSE sequence included the following parameters: slice thickness, 4 mm; 24 slices; intersection gap, 1 mm; TR/TE, 4,000 ms/100 ms; matrix 320×224; FOV, 240×240 mm; and two signals acquired, covering the larynx. After gadolinium injection, T1-weighted fat-saturated sequences were performed in the axial plane (using identical parameters to precontrast medium administration) and in the coronal plane (24 slices; slice thickness, 4 mm; intersection gap, 1 mm; TR/TE, 540 ms/9.2 ms; matrix, 320×224; FOV, 240×220 mm; and two signals acquired, fat suppression).

DWI with TSE techniques was performed at the same section position as the axial and T1-weighted images. The parameters were as follows: TR/TE  = 8,000 ms/60 ms; FOV 240×240 cm; matrix, 124×124; 24 slices; slice thickness, 4 mm; and b  = 0 or 1,000 s/mm^2^). ADC maps were generated with Extended MR Workspace (EWS). A short-tau inversion recovery (STIR) sequence was used for fat suppression in the diffusion-weighted sequence.

### 4. Analysis of the MR Images

The imaging data were reviewed by two radiologists with no knowledge of the primary lesion; they reached a consensus opinion before reviewing the pathology results. The lesion contour, size, and internal architecture were documented. An ADC map was generated by DWI with EWS, and the ADC value was measured. All laryngeal lesions were characterized based on the signal intensity on T1- and T2-weighted MR images and enhancement characteristics. Tumor-volume measurements were performed by one reviewer (DS Shang) on contrast-enhanced axial T1-weighted images using a computerized image-analysis tool that is available as part of the PACS at our hospital. DW-MRI at a native b value of 1,000 s/mm^2^ (b-1,000 images) and the corresponding ADC maps were matched to and evaluated with the morphological images as previously described [20]. A hyperintense signal on the native b-1,000 image compared with the surrounding tissue with corresponding low signal intensity in the matching ADC map was considered positive for a tumor. A high signal intensity on b-1,000 images with a corresponding high signal on the matching ADC map was considered to represent T2 shine-through and, therefore, the absence of a tumor. The absence of hyperintensity on the b-1,000 image was also considered negative for a tumor. The region of interest (ROI) was placed by a single radiologist (DS Shang) with 15 years of experience in the interpretation of body MR images to avoid interobserver bias. ROIs were placed within the solid part of the tumor. The ROI was placed on the native DWI using T1-weighted, T2-weighted, or contrast-enhanced T1-weighted images as reference images. If a lesion was superficial and small, the ADCs of both vocal cords were delineated. ROIs were not positioned in the cystic or necrotic portion identified on the T2-weighted images and the contrast enhanced T1-weighted images because this might influence the quantitative data. The mean±standard deviation (SD) of the ADC values for the laryngeal lesions was calculated.

### 5. Statistical Analyses

Statistical analyses were performed using SPSS software (ver. 19.0 for Windows; SPSS Inc., Chicago, IL, USA). P-values <0.05 were considered to indicate statistical significance. Differences in size and the ADCs of the laryngeal lesions between patients with malignant and precancerous lesions were tested with an independent samples *t*-test.

Receiver-operating-characteristic (ROC) curve analysis was used to investigate the discriminatory capability of the ADCs in differentiating laryngeal carcinomas from laryngeal precancerous lesions. The area under the ROC curve was calculated. The ADC value that corresponded to the highest Yoden index (sensitivity+specificity - 1) was chosen as the optimal ADC threshold value because it optimized both the sensitivity and specificity. We measured the interrater agreement using intraclass correlation coefficients (ICC). ICC values ranged between 0 and 1, with higher ICC values indicating stronger agreement. ICCs were classified according to a previously described method [26]. ICCs >0.80 are reliable for basic research, and ICCs >0.90 are necessary for essential assessments of individuals in the clinic [26].

## Results

### 1. Clinical Characteristics of the Patients

The mean age of the patients with laryngeal precancerous lesions was 63.7 years (49–80 years). The symptoms included hoarseness and pharyngalgia. All 33 laryngeal carcinomas were SCCs. Twenty-four (72.7%) and nine (27.3%) patients had tumors located in the glottis and supraglotti, respectively. There were no patients with tumors in the subglottis. According to the International Union Against Cancer TNM classification system (2007, 7^th^ edition), 18 (54.5%) patients were stage T_1_N_0_M_0_, 11 (33.3%) were stage T_2_N_0_M_0_, 1 (3.0%) was stage T_1_N_1_M_0_, 1 (3.0%) was stage T_1_N_2_M_0_, and 2 (6.1%) were stage T_2_N_2_M_0._ Among the 17 precancerous lesions, there were 7 cases of moderate dysplasia and 9 of severe dysplasia and carcinoma *in situ*.

### 2. Laryngostroboscopy in Discriminating Laryngeal Lesions

Of 33 laryngeal carcinoma patients, 27 were diagnosed by laryngostroboscopy. Of 17 laryngeal precancerous patients, 6 were diagnosed with laryngeal carcinomas by laryngostroboscopy. The sensitivity, specificity, and accuracy were 81.8%, 64.7%, 76.0% respectively.

#### Conventional MR images and DWI of laryngeal lesions

Conventional MR images showed that 30 of 33 pathologically proven laryngeal carcinomas were diagnosed as laryngeal carcinomas, and 13 of 17 pathologically proven laryngeal precancerous lesions were diagnosed as precancerous lesions. The sensitivity, specificity, and accuracy were 90.9%, 76.5%, and 86.0%, respectively.

**Figure 1:**
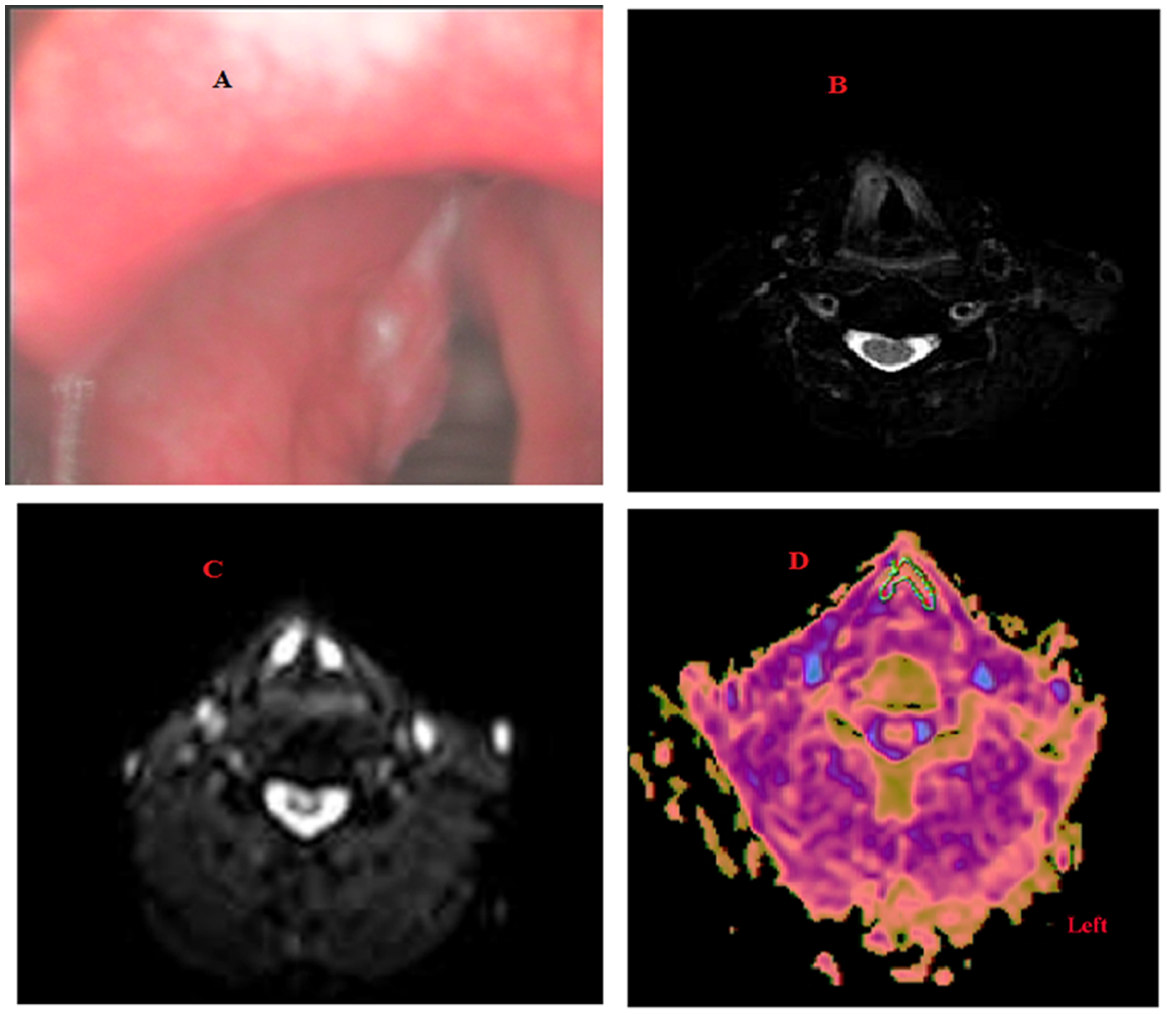
A patient with pathologically proven T_1b_N_0_M_0_ laryngeal carcinoma. A: A lesion was only found in the right vocal cord, and another lesion in the left vocal cord was not found by laryngostroboscopy. B: Transverse T2-weighted MRI showed a tumor in the right vocal cord. C: DWI suggested lesions were in the bilateral vocal cords (b = 1000 s/mm^2^). D: Consequently, the corresponding ADC map revealed hypointense lesions in the bilateral cord (ADC = 1.05×10^−3^ mm^2^/s in the left vocal cord, ADC = 0.90×10^−3^ mm^2^/s in the right vocal cord).

**Figure 2:**
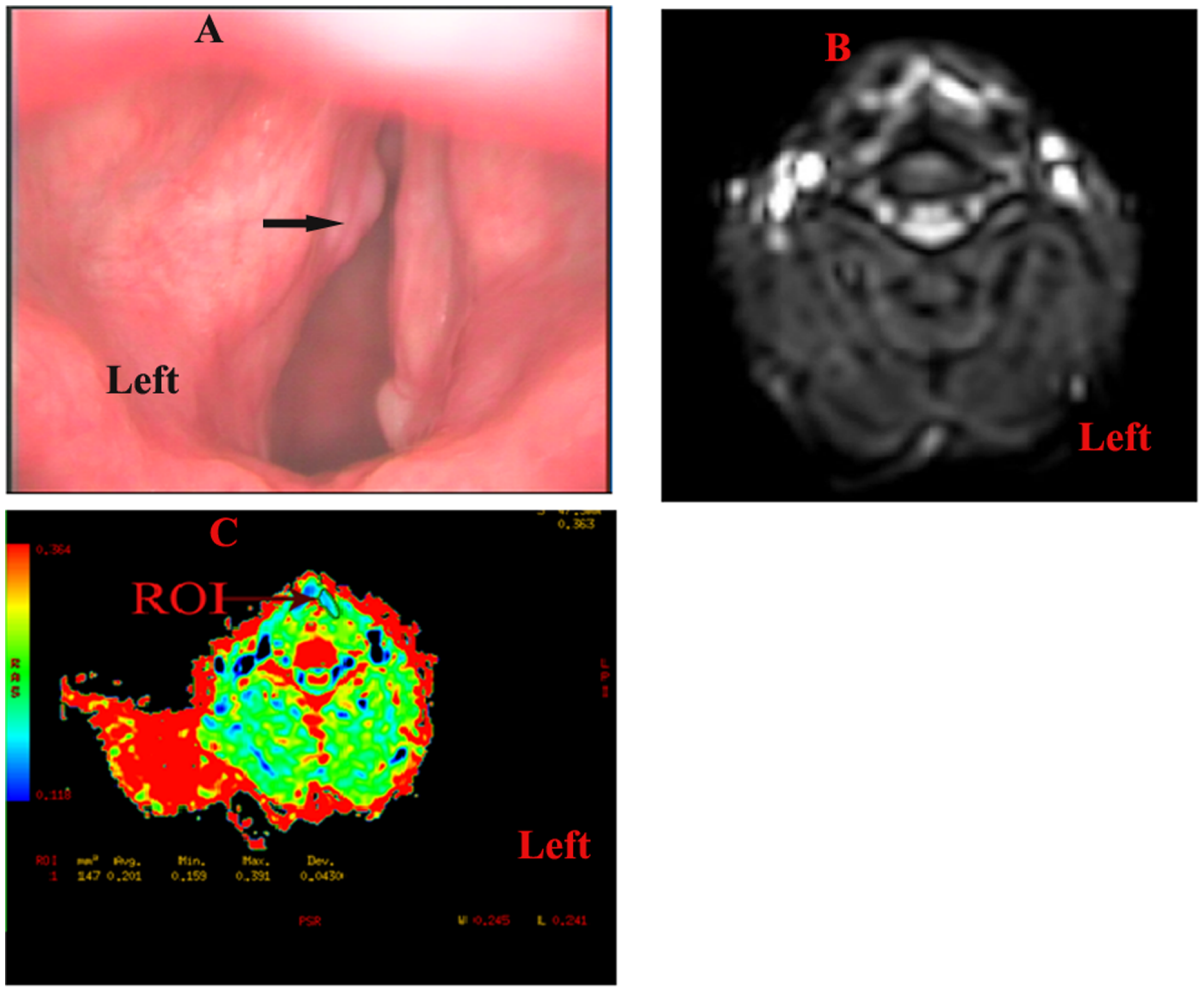
A patient with a pathologically proven laryngeal precancerous lesion. A: A lesion in the left vocal cord was suspected to be laryngeal carcinoma by laryngostroboscopy. B: DWI suggested hyperintense lesions in the left vocal cords (b = 1000 s/mm^2^). C: The corresponding ADC map also revealed hyperintense lesions in the left cord (ADC = 2.01×10^−3^ mm^2^/s). These findings suggest that the lesion in the left vocal cord may be benign.

Using qualitative DWI analysis, we could discriminate all laryngeal carcinomas from precancerous lesions, but DWI did not show two patients with precancerous lesions, resulting in two false-positive results. The sensitivity, specificity, and accuracy were 100.0, 88.2, and 96.0%, respectively. There was no significant difference between the sensitivity, specificity, and accuracy of laryngoscopy and DWI (P>0.05). A lesion was only found in the right vocal cord, and another lesion in the left vocal cord was not found by laryngostroboscopy. DWI accurately suggested lesions were in the bilateral vocal cords with pathologically proven T_1b_N_0_M_0_ (Fig. 1). A patient with pathologically proven laryngeal mild dysplasia was suspected of having laryngeal carcinoma in the left vocal cord by laryngostroboscopy, and DWI accurately suggested the lesion was benign (Fig. 2).

### 3. Diagnostic Ability of ADC Values at b  = 1,000 s/mm^2^ in Laryngeal Lesions

**Figure 3:**
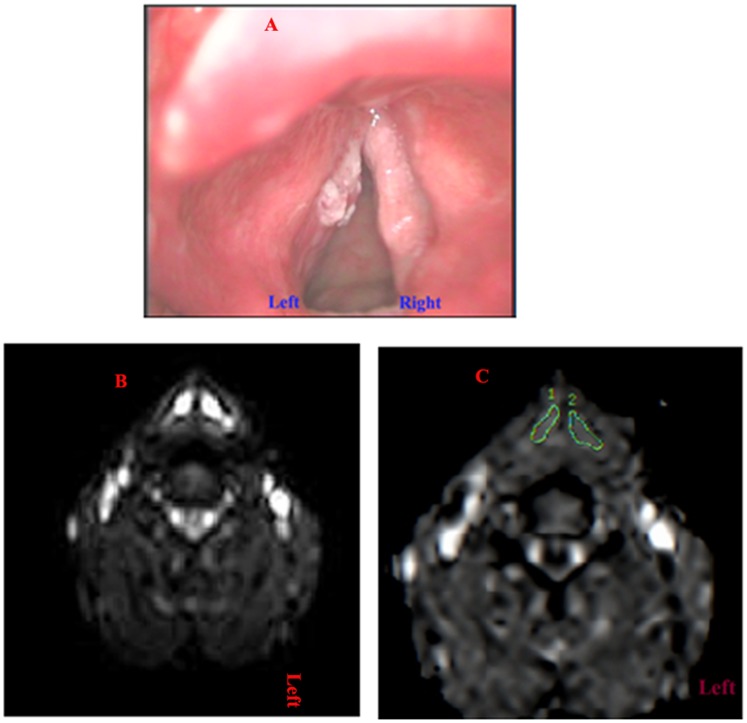
A patient with pathologically proven laryngeal carcinoma in the left vocal cord and mild dysplasia in the right vocal cord. A: Laryngostroboscopy showed a 5 × 6-mm mass in the left vocal cord and rough mucosa in the anterior right vocal cord suspected to be bilateral laryngeal carcinoma in the vocal cords. B: DWI-suggested lesions were identified in the bilateral vocal cords (b = 1000 s/mm^2^). C: Consequently, the corresponding ADC map reveals hypointense of lesions in the left vocal cord. (ADC = 1.26×10^−3^ mm^2^/s), and hyperintense of lesions in the right vocal cord(ADC = 1.67×10^−3^ mm^2^/s).

**Figure 4:**
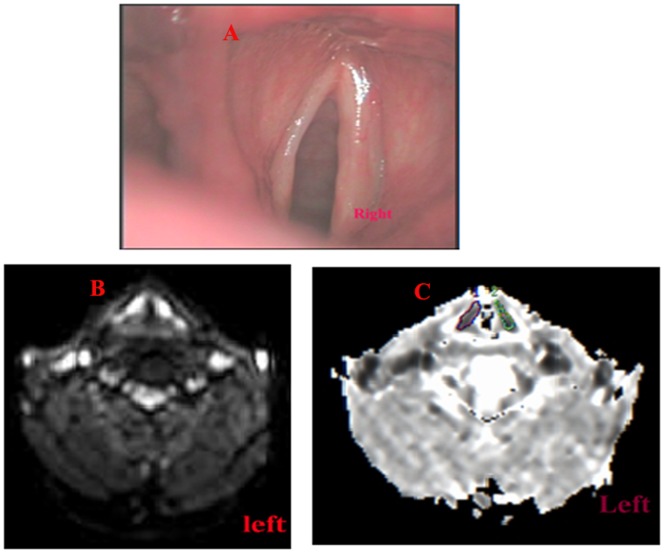
A patient with pathologically proven laryngeal carcinoma in the left vocal cord. A: A small superficial lesion in the left vocal cord was found by laryngostroboscopy. B: DWI suggested a lesion in the left vocal cord to be hyperintense. C: Consequently, the ADCs of both vocal cords were delineated (ADC = 1.40×10^−3^ mm^2^/s in the left vocal cord, ADC = 1.65×10^−3^ mm^2^/s in the right vocal cord). These findings suggest that the lesion in the left vocal cord may be malignant, and that in the right vocal cord may be benign.

**Figure 5:**
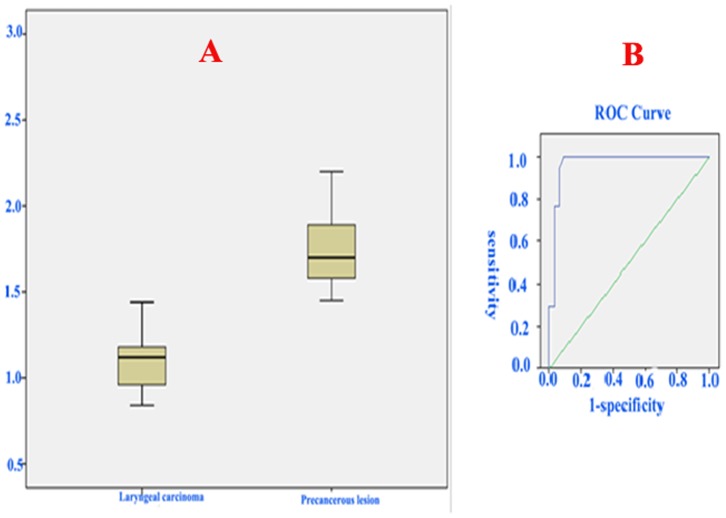
A: Box and whisker plots showing the ADC values of laryngeal carcinomas (LCs) and precancerous lesions (PLs). The ADC values were lower for patients with laryngeal carcinoma (mean 1.195±0.32×10^−3^ mm^2^/s) compared with those with laryngeal precancerous lesions (mean 1.780±0.32×10^−3^ mm^2^/s; *P*<0.001). B: Receiver operating characteristic (ROC) analysis showed that the area under the curve was 0.956, whereas the optimal threshold for the ADC was 1.455×10^−3^ mm^2^/s.

For interobserver agreements of ADC values in lesions on laryngeal carcinomas and precancerous lesions, ICCs of 0.90 and 0.92 respectively. The ADC values were lower for patients with laryngeal carcinoma (mean 1.195±0.32×10^−3^ mm^2^/s) compared with those with laryngeal precancerous lesions (mean 1.780±0.32×10^−3^ mm^2^/s; P<0.001) (Fig. 3,4). ROC analysis showed that the area under the curve was 0.956 while the optimal threshold for the ADC was 1.455×10^−3^ mm^2^/s, resulting in a sensitivity of 94.1%, a specificity of 90.9%, and an accuracy of 92.9% (Fig. 5).

## Discussion

The structure of the larynx is complex, with many different tissues, including mucosa, cartilage (ossified or non-ossified), muscle, fat, and air in close proximity, and various physiological movements, including swallowing, breathing, speaking, and impulses arising from major blood vessels. These issues result in distortion and failed fat suppression artifacts that can cause non-diagnostic images. Because of these limitations, there was been little use of DWI in the head and neck. To date, there have been few reports about DWI in laryngeal lesions[20]–[23]. Most have reported value in detecting recurrent tumors after radiotherapy/chemotherapy [20], [22], [23]. The use of DWI to discriminate malignant from benign lesions in the head and neck has been investigated in a limited number of studies; however, there is no previous report about the preoperative discrimination of laryngeal carcinomas from precancerous lesions.

These limitations have gradually been overcome with steady improvements in MRI techniques [15], [19], [20] driven by a wide variety of potential applications [19]. DWI of the head and neck has commonly been done with a 1.5-T scanner. The signal gain at 3.0 T improves imaging of the head and neck with respect to spatial resolution and acquisition time. In this preliminary study, we found that conventional MRI may distinguish laryngeal carcinomas and precancerous lesions with 3.0-T MR. Adding qualitative DWI analysis, we could discriminate all laryngeal carcinomas from precancerous lesions. The sensitivity, specificity, and accuracy were 100.0, 88.2, and 96.0%, respectively. Although the diagnosis was not improved statistically compared with laryngostroboscopy, DWI may detect changes in tumor size and shape before they are visible to the naked eye. Laryngostroboscopy has an advantage in judging motion in the larynx, but it does not detect changes under the mucosa or the multifocal nature of tumors. In the present study, a lesion was found in the right vocal cord and another lesion in the left vocal cord in a patient with pathologically proven T_1b_N_0_M_0_ by laryngostroboscopy. DWI accurately suggested that the lesions were in bilateral vocal cords. Accurate preoperative diagnoses and T staging may help direct the most appropriate management.

Several studies have already shown the benefit of DWI in distinguishing malignant and benign tumors in the head and neck [9], [10], [15], [28]–[30]. These abilities are always aided by b-values and quantitative ADC values. The ADC value varies strongly with the underlying b-values chosen. In theory, hypercellular tumor tissue leads to impeded diffusion and, subsequently, a lower ADC value [9]. Thus, malignant tumors usually show lower ADC values than benign tumors [10]. This was supported by Eida et al. [28], who reported that the ADC was significantly greater in benign salivary tumors than in malignant salivary tumors preoperatively. They also found that the ADCs (using b-values of 500 and 1,000 s/mm^2^) of sinonasal malignant tumors were significantly lower than those of benign tumors [29]. Srinivasan et al. [15] reported that there was a significant difference between the mean ADC values in benign and malignant lesions of the head and neck. Wang et al. [30] showed a significantly smaller ADC for malignant lesions, including SCC, than for benign lesions, in the head and neck.

The results of this study are similar to those of previous reports. The ADC values were lower for patients with laryngeal carcinomas (mean 1.195±0.32×10^−3^ mm^2^/s) compared with those with laryngeal precancerous lesions (mean 1.780±0.32×10^−3^ mm^2^/s; P<0.001). The ADC cutoff in our preliminary study was 1.455×10^−3^ mm^2^/s, resulting in a sensitivity of 94.1%, a specificity of 90.9%, and an accuracy of 92.9%. This threshold did not markedly overlap for laryngeal carcinomas and precancerous lesions. ROC analysis showed that the area under the curve was 0.956. These findings suggest that an ADC threshold of 1.455×10^−3^ mm^2^/s is optimal for distinguishing laryngeal carcinomas and precancerous lesions. However, there is not a standard ADC threshold in the head and neck region for differentiating malignant lesions from benign lesions. The threshold point varies between different parts of the head and neck and from one study to another [10]. In other studies [15], [20], [25], [29], [30], the ADC threshold for discriminating between malignant tumors and benign tumors was lower than ours. Srinivasan et al. [15] established an optimal ADC threshold of 1.3×10^−3^ mm^2^/s for diagnosis of lesions in the head and neck (Using that value in the present study, the sensitivity, specificity, and accuracy were 100, 75, and 92%, respectively). Abdel Razek et al. [25]found that an ADC of 1.25×10^−3^ mm^2^/s was useful as a threshold for differentiating malignant from benign head and neck masses (Using that value in the present study, the sensitivity, specificity, and accuracy in were 100, 73, and 92%, respectively). Sasaki et al. [29] reported that a lower ADC cutoff of 0.84×10^−3^ mm^2^/s was best for differentiating sinonasal benign/inflammatory lesions from malignant tumors; its sensitivity, specificity, and accuracy were 61, 94, and 79%, respectively (Using that value in the present study, the sensitivity, specificity, and accuracy were 100%, only 3%, and 92%, respectively). An ADC value of 1.22×10^−3^ mm^2^/s was found to be the optimal threshold for differentiating between benign and malignant tumors in the head and neck and had 84% sensitivity, 91% specificity, and 87% accuracy [30] (Using that value the present study, the sensitivity, specificity, and accuracy were 100, 70, and 92%, respectively). These results showed that there was a larger overlap between laryngeal carcinoma and precancerous lesions if these previously reported thresholds were used in the present study. To our knowledge, there is one previous report on DWI in laryngeal carcinoma [23]. DWI was used in only four laryngeal carcinomas after radiotherapy, where a lower ADC in tumor recurrence than in benign post-therapeutic alterations was found [23]. In one study of 46 laryngeal and hypopharyngeal cancers after chemoradiotherapy, although bi-exponential fitting (ADC_D_ and F_P_) was performed to interpret the results of DWI, Tshering Vogel et al. [20] reported that 1.30×10^−3^ mm^2^/s was the optimal ADC cutoff for differentiating tumor recurrence after chemoradiotherapy from non-tumor changes. However, they found a larger overlap between benign and malignant outcomes. They suggested that the cause may have been that the larynx is subject to more lead susceptibility and movement artifacts than other locations in the head and neck, and the ratio of T_1_ patients in their study was relatively large, so the sizes of the tumors were smaller. An echo-planar-based sequence (EPI) is the traditional choice for DWI. EP sequences are very fast but are sensitive to susceptibility artifacts and image distortions, which are particularly present at air-tissue and bone-tissue interfaces. EPI DWI is an easier imaging modality that is sensitive to susceptibility artifacts in laryngeal diseases. Some reports have demonstrated that a turbo spin-echo (TSE) DW sequence does not suffer from susceptibility artifacts. The use of a relatively high spatial resolution may allow the detection of small lesions [19], [25]–[27].

In the present study, although 20 of 33 laryngeal carcinomas were of T_1_ stage, the overlap between the laryngeal carcinomas and laryngeal precancerous lesions was smaller than in the above study. In the initial stage, DWI also readily produces susceptibility artifacts and six patients with susceptibility artifacts were excluded from our study. Next, we introduced a TSE-based DW sequence with 3.0 T MR instead of an echo-planar sequence, and we encouraged the patients not to swallow, speak, or cough during imaging. In addition, we took thin slices, applied meticulous shimming, and used sight fixation of the patient’s head to minimize susceptibility artifacts. Our results demonstrate the potential value of this technique in the evaluation of laryngeal carcinomas.MRI was performed on a 1.5-T unit in the study of Tshering Vogel et al. [20]. In the prostate, Rylander et al. [31] found that artifact volumes were lower with a 3.0-T MRI scanner than with a 1.5-T machine. Srinivasan et al. [15] used a 3.0-T MR unit with b-values of 0 and 800 s/mm^2^ to differentiate benign and malignant lesions in the head and neck. They suggested that at 3 T, MRI with a multichannel coil can achieve small, 4-mm sections, shorter echo times (45 ms), and smaller in-plane voxel sizes, which can be used to decrease susceptibility artifacts [10], [15]. The ADC cutoff in our study was also higher (1.455×10^−3^ mm^2^/s) using b-values of 0 and 1,000 s/mm^2^ than in the study by Tshering Vogel et al. [20] (1.30×10^−3^ mm^2^/s).

Our study has several limitations. First, the study was conducted using a single-center registry of patients with laryngeal carcinoma, and the number of patients was small. Thus, additional studies including greater numbers of patients with preoperative laryngeal carcinoma are needed to confirm the results of our preliminary study. Additionally, patients with laryngeal carcinomas were not stratified by T stage in this study. Specifically, laryngeal carcinomas at the T1 stage may affect the accuracy of the ADC because for tumors at this stage air-tissue artifacts are easily produced. Future improvements in MRI techniques and equipment will further reduce susceptibility effects.

### Conclusions

Despite its limitations, including the small number of laryngeal carcinomas included, DWI may be useful to detect changes in tumor size and shape before they are visible by laryngostroboscopy. The ADC values were lower for patients with laryngeal carcinomas than for those with laryngeal precancerous lesions. The ADC threshold in this preliminary study was 1.455×10^−3^ mm^2^/s, resulting in a sensitivity of 94.1%, a specificity of 90.9%, and an accuracy of 92.9%. This threshold did not overlap markedly for laryngeal carcinomas and precancerous lesions.

## References

[1] Gallo O, Bianchi S, Giannini A, Boccuzzi S, Calzolari A, et al (1994) Lack of detection of human papillomavirus (HPV) in transformed laryngeal keratoses by in situ hybridization (ISH) technique. Acta Otolaryngol 114: 213–217. 8203204 10.3109/00016489409126045

[2] Warnecke A, Averbeck T, Leinung M, Soudah B, Wenzel GI, et al (2010) Contact endoscopy for the evaluation of the pharyngeal and laryngeal mucosa. Laryngoscope 120: 253–258. 19998420 10.1002/lary.20732

[3] Kraft M, Betz CS, Leunig A, Arens C (2011) Value of fluorescence endoscopy for the early diagnosis of laryngeal cancer and its precursor lesions. Head Neck 33: 941–948. 21674669 10.1002/hed.21565

[4] Malzahn K, Dreyer T, Glanz H, Arens C (2002) Autofluorescence endoscopy in the diagnosis of early laryngeal cancer and its precursor lesions. Laryngoscope 112: 488–493. 12148859 10.1097/00005537-200203000-00015

[5] Hu Q, Zhu SY, Zhang Z, Luo F, Mao YP, et al (2011) Assessment of glottic squamous cell carcinoma: comparison of sonography and non-contrast-enhanced magnetic resonance imaging. J Ultrasound Med 30: 1467–1474. 22039019 10.7863/jum.2011.30.11.1467

[6] Becker M, Zbären P, Casselman JW, Kohler R, Dulguerov P, et al (2008) Neoplastic invasion of laryngeal cartilage: reassessment of criteria for diagnosis at MR imaging. Radiology. 249: 551–559. 10.1148/radiol.2492072183 18936314

[7] Kim JW, Yoon SY, Park IS, Park SW, Kim YM (2008) Correlation between radiological images and pathological results in supraglottic cancer. J Laryngol Otol 122: 1224–1229. 18312707 10.1017/S0022215108001746

[8] Becker M, Burkhardt K, Dulguerov P, Allal A (2008) Imaging of the larynx and hypopharynx. Eur J Radiol 66: 460–479. 18495402 10.1016/j.ejrad.2008.03.027

[9] Thoeny HC (2011) Diffusion-weighted MRI in head and neck radiology: applications in oncology. Cancer Imaging 10: 209–214. 21317090 10.1102/1470-7330.2010.0030 PMC3080114

[10] Razek AA (2010) Diffusion-weighted magnetic resonance imaging of head and neck. J Comput Assist Tomogr 34: 808–815. 21084893 10.1097/RCT.0b013e3181f01796

[11] Vandecaveye V, De Keyzer F, Dirix P, Lambrecht M, Nuyts S, et al (2010) Applications of diffusion-weighted magnetic resonance imaging in head and neck squamous cell carcinoma. Neuroradiology 52: 773–784. 20631998 10.1007/s00234-010-0743-0

[12] Perrone A, Guerrisi P, Izzo L, D’Angeli I, Sassi S, et al (2011) Diffusion-weighted MRI in cervical lymph nodes: differentiation between benign and malignant lesions. Eur J Radiol 77: 281–286. 19716671 10.1016/j.ejrad.2009.07.039

[13] Holzapfel K, Duetsch S, Fauser C, Eiber M, Rummeny EJ, et al (2009) Value of diffusion-weighted MR imaging in the differentiation between benign and malignant cervical lymph nodes. Eur J Radiol 72: 381–387. 18995981 10.1016/j.ejrad.2008.09.034

[14] Razek AA, Megahed AS, Denewer A, Motamed A, Tawfik A, et al (2008) Role of diffusion-weighted magnetic resonance imaging in differentiation between the viable and necrotic parts of head and neck tumors. Acta Radiol 49: 364–370. 18365828 10.1080/02841850701777390

[15] Srinivasan A, Dvorak R, Perni K, Rohrer S, Mukherji SK (2008) Differentiation of benign and malignant pathology in the head and neck using 3T apparent diffusion coefficient values: early experience. AJNR Am J Neuroradiol 29: 40–44. 17921228 10.3174/ajnr.A0743 PMC8119114

[16] Abdel Razek AA, Kandeel AY, Soliman N, El-shenshawy HM, Kamel Y, et al (2007) Role of diffusion-weighted echo-planar MR imaging in differentiation of residual or recurrent head and neck tumors and posttreatment changes. AJNR Am J Neuroradiol 28: 1146–1152. 17569975 10.3174/ajnr.A0491 PMC8134145

[17] Vandecaveye V, Dirix P, De Keyzer F, Op de Beeck K, Vander Poorten V, et al (2012) Diffusion-weighted magnetic resonance imaging early after chemoradiotherapy to monitor treatment response in head-and-neck squamous cell carcinoma. Int J Radiat Oncol Biol Phys 82: 1098–1107. 21514067 10.1016/j.ijrobp.2011.02.044

[18] Galbán S, Lemasson B, Williams TM, Li F, Heist KA, Johnson TD, et al (2012) DW-MRI as a biomarker to compare therapeutic outcomes in radiotherapy regimens incorporating temozolomide or gemcitabine in glioblastoma. PLoS One 7: e35857. 22536446 10.1371/journal.pone.0035857 PMC3334987

[19] Thoeny HC, De Keyzer F, King AD (2012) Diffusion-weighted MR imaging in the head and neck. Radiology 263: 19–32. 22438440 10.1148/radiol.11101821

[20] Tshering Vogel DW, Zbaeren P, Geretschlaeger A, Vermathen P, De Keyzer F, et al (2013) Diffusion-weighted MR imaging including bi-exponential fitting for the detection of recurrent or residual tumour after (chemo)radiotherapy for laryngeal and hypopharyngeal cancers. Eur Radio 23: 562–569. 10.1007/s00330-012-2596-x 22865270

[21] Hatakenaka M, Nakamura K, Yabuuchi H, Shioyama Y, Matsuo Y, et al (2011) Pretreatment apparent diffusion coefficient of the primary lesion correlates with local failure in head-and-neck cancer treated with chemoradiotherapy or radiotherapy. Int J Radiat Oncol Biol Phys 81: 339–345. 20832179 10.1016/j.ijrobp.2010.05.051

[22] de Bree R, van der Putten L, Brouwer J, Castelijns JA, Hoekstra OS, et al (2009) Detection of locoregional recurrent head and neck cancer after (chemo)radiotherapy using modern imaging. Oral Oncol 45: 386–393. 19095487 10.1016/j.oraloncology.2008.10.015

[23] Vandecaveye V, de Keyzer F, Vander Poorten V, Deraedt K, Alaerts H, et al (2006) Evaluation of the larynx for tumour recurrence by diffusion-weighted MRI after radiotherapy: initial experience in four cases. Br J Radiol 79: 681–687. 16641411 10.1259/bjr/89661809

[24] Abdel Razek AA, Gaballa G, Elhawarey G, Megahed AS, Hafez M, et al (2009) Characterization of pediatric head and neck masses with diffusion-weighted MR imaging. Eur Radiol 19: 201–208. 18704436 10.1007/s00330-008-1123-6

[25] de Graaf P, Pouwels PJ, Rodjan F, Moll AC, Imhof SM, et al (2012) Single-shot turbo spin-echo diffusion-weighted imaging for retinoblastoma: initial experience. AJNR Am J Neuroradiol 33: 110–118. 22033715 10.3174/ajnr.A2729 PMC7966176

[26] Verhappen MH, Pouwels PJ, Ljumanovic R, van der Putten L, Knol DL, et al (2012) Diffusion-weighted MR imaging in head and neck cancer: comparison between half-fourier acquired single-shot turbo spin-echo and EPI techniques. AJNR Am J Neuroradiol 33: 1239–1246. 22322615 10.3174/ajnr.A2949 PMC7965518

[27] Sakamoto J, Sasaki Y, Otonari-Yamamoto M, Sano T (2012) Comparison of various methods for quantification of apparent diffusion coefficient of head and neck lesions with HASTE diffusion-weighted MR imaging. Oral Surg Oral Med Oral Pathol Oral Radiol 114: 266–276. 22769413 10.1016/j.oooo.2012.03.015

[28] Eida S, Sumi M, Sakihama N, Takahashi H, Nakamura T (2007) Apparent diffusion coefficient mapping of salivary gland tumors: prediction of the benignancy and malignancy. AJNR Am J Neuroradiol 28: 116–121. 17213436 PMC8134115

[29] Sasaki M, Eida S, Sumi M, Nakamura T (2011) Apparent diffusion coefficient mapping for sinonasal diseases: differentiation of benign and malignant lesions. AJNR Am J Neuroradiol 32: 1100–1106. 21393402 10.3174/ajnr.A2434 PMC8013129

[30] Wang J, Takashima S, Takayama F, Kawakami S, Saito A, et al (2001) Head and neck lesions: characterization with diffusion-weighted echo-planar MR imaging. Radiology 220: 621–630. 11526259 10.1148/radiol.2202010063

[31] Rylander S, Thörnqvist S, Haack S, Pedersen EM, Muren LP (2011) Intensity profile based measurement of prostate gold markers influence on 1.5 and 3T diffusion-weighted MR images. Acta Oncol 50: 866–872. 21767186 10.3109/0284186X.2011.590523

